# Does Sex-Selective Predation Stabilize or Destabilize Predator-Prey Dynamics?

**DOI:** 10.1371/journal.pone.0002687

**Published:** 2008-07-16

**Authors:** David S. Boukal, Luděk Berec, Vlastimil Křivan

**Affiliations:** 1 Institute of Marine Research, Bergen, Norway; 2 Department of Biology, University of Bergen, Bergen, Norway; 3 Department of Theoretical Ecology, Institute of Entomology, Biology Centre AS CR, České Budějovice, Czech Republic; The University of New South Wales, Australia

## Abstract

**Background:**

Little is known about the impact of prey sexual dimorphism on predator-prey dynamics and the impact of sex-selective harvesting and trophy hunting on long-term stability of exploited populations.

**Methodology and Principal Findings:**

We review the quantitative evidence for sex-selective predation and study its long-term consequences using several simple predator-prey models. These models can be also interpreted in terms of feedback between harvesting effort and population size of the harvested species under open-access exploitation. Among the 81 predator-prey pairs found in the literature, male bias in predation is 2.3 times as common as female bias. We show that long-term effects of sex-selective predation depend on the interplay of predation bias and prey mating system. Predation on the ‘less limiting’ prey sex can yield a stable predator-prey equilibrium, while predation on the other sex usually destabilizes the dynamics and promotes population collapses. For prey mating systems that we consider, males are less limiting except for polyandry and polyandrogyny, and male-biased predation alone on such prey can stabilize otherwise unstable dynamics. On the contrary, our results suggest that female-biased predation on polygynous, polygynandrous or monogamous prey requires other stabilizing mechanisms to persist.

**Conclusions and Significance:**

Our modelling results suggest that the observed skew towards male-biased predation might reflect, in addition to sexual selection, the evolutionary history of predator-prey interactions. More focus on these phenomena can yield additional and interesting insights as to which mechanisms maintain the persistence of predator-prey pairs over ecological and evolutionary timescales. Our results can also have implications for long-term sustainability of harvesting and trophy hunting of sexually dimorphic species.

## Introduction

Mechanisms promoting persistence and stability of food webs represent a fundamental challenge in ecology. Many species reproduce sexually, yet we know little about potential implications of different male and female life histories on population dynamics and food web interactions [1]. There are, however, at least two reasons why the distinction between males and females can be important in food web dynamics.

First, sex-selective predation should be a widespread phenomenon. Many prey species exhibit sexual dimorphism in appearance, physiology and behaviour, while predators often prefer prey with certain size, conspicuousness, morphology or habits [2]–[4]. Sex bias in predation will be determined by the nature of the prey's sexual dimorphism and the predator's preferences and foraging tactics. Male-biased predation is frequently related to prey traits shaped by sexual selection [3], [5]. Males are usually more active than females [6] and numerous studies have demonstrated that predators and parasitoids are attracted by mating signals of their male prey [3] and references therein. Males are also often more conspicuous [7] and the exaggerated secondary traits may impair their predator-avoidance behaviour e.g., [8]. On the other hand, female-biased predation is often related to prey traits shaped by fecundity selection. Females are often larger, which can make them easier to detect or more rewarding as prey e.g., [9]. They can also suffer from increased predation during the reproductive period, usually because of activities related to parenting duties [10], [11], and references therein. However, reports of sex-selective predation largely come from anecdotal observations and short-term experiments [3] and references therein. None of the empirical studies tried to evaluate population consequences of sex-selective predation, and we thus have no clear understanding of its long-term impacts. In many exploited species, males and females are also harvested at different rates, either because one of the sexes is easier to capture [12] or more valuable [13]. The impact of sex-selective harvest on the dynamics of exploited species is poorly understood as well.

Second, male- and female-biased predation can impact population dynamics differently; the net result will be a combination of direct effects due to reduced male and female densities in the prey and indirect effects due to apparent competition between both sexes of the prey mediated by the shared predator. Previous models showed that population dynamics of sexually reproducing species are shaped by the mating system and, consequently, by the reproductive success of individual females [14]. The presence of males will affect reproductive rate, equilibrium population densities [1] and their stability [14]–[16]. If female mating rate decreases at low male numbers or densities, this will lead to positive density dependence in the per-capita population growth rate—the mate finding Allee effect [17]–[19]. Models show that Allee effects can destabilize predator-prey dynamics and that such systems often collapse [20]–[22]. An anthropogenic Allee effect due to disproportionately high prices of rare exploited species can lead to their extinction [23]. However, none of these models considered male and female prey separately.

In this paper we combine a literature review with a theoretical modelling approach to investigate predator-prey systems in which predators capitalize on sexual dimorphism in behaviour, morphology and/or physiology of the prey species [3], [5], [9]. Our model can also describe dynamics of an exploited species in which the sexes are harvested at different rates, extending the model studied in [23]. Throughout the paper, all issues related to males, females and sex-specificity in general always pertain to the prey. We first summarize empirical data on sex-selective predation in the literature to quantify predation biases towards either sex of the prey. Using a simple model, we then aim at answering the following questions: Can sex-selective predation alone stabilize predator-prey dynamics? How are the (de)stabilizing properties of male- or female-biased predation linked to the prey mating system? How do the mate-finding Allee effect and other (de)stabilizing mechanisms influence the results? Finally, we link the review of sex-selective predation with our theoretical study and discuss how the observed prevalence of male-biased predation can relate to our modelling results, what implications our results can have for exploited species, and highlight several promising directions for future research.

## Methods

We searched for studies that report differences between male and female predation mortality within the Web of Science and Zoological Record databases, including some secondary references. Since none of the studies reporting sex-selective predation focused on population dynamics, we also examine a simple extension of the classical Lotka-Volterra predator-prey model to expose the consequences of sex-selective predation for predator-prey dynamics.

The model distinguishes between male (*m*) and female (*f*) prey and unstructured predator (*x*) populations. It accounts for a range of prey mating systems and can include a mate-finding Allee effect in the prey:
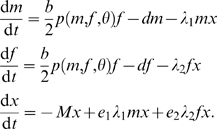
(1)


We assume that the prey sex ratio at birth is unbiased, the intrinsic mortality rate *d* is equal in male and female prey, and the birth rate *b* per female prey in the absence of mating constraints is sufficiently high (*b*>2*d*) such that the prey population has positive growth rate in the absence of predation and Allee effects. Parameters *λ*_i_ scale the linear sex-specific functional responses of the predator to male and female prey, *e*_i_ denote the efficiencies with which consumed male and female prey are converted into new predators, and *M* is the predator per-capita mortality rate. The maximum prey birth rate is scaled by *p*(*m*,*f*,θ), which is the female mating rate or the probability that a female becomes fertilized per unit time [18], [26].

**Figure 1:**
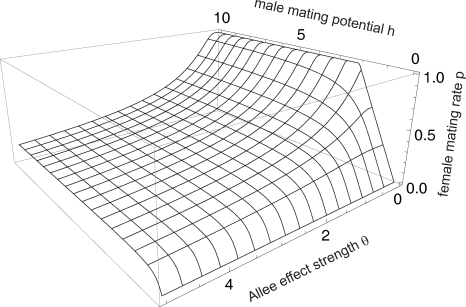
Shape of mating function (3). The mating function increases in *h*, decreases in *θ*, and reduces to *p*(*m*,*f*) = min(*hm*/*f*,1) in the absence of the mate-finding Allee effect (*θ* = 0). Male and female population sizes in the figure: *m* = 1, *f* = 2.

Function *p* incorporates both the mate-finding Allee effect in the prey (through parameter θ≥0) and the prey mating system. If mating opportunities are unlimited, *p* = 1. For the mate-finding Allee effect and unlimited male mating potential, the female mating rate can be described by the negative exponential function of male density [17], [18]

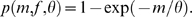
(2)We refer to this mating function as unlimited polygyny (Table 1). Constraints on male mating potential or social system that lead to ‘limited’ polygyny, monogamy or polyandry can be described as
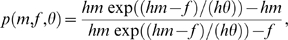
(3)in which *h* represents, depending on the mating system, the number of matings a male can achieve with different females per unit time or a male's harem size (Fig. 1). Values of *h*>1 correspond to limited polygyny (including polygynandry in the sense of Shuster and Wade [27]), *h* = 1 to monogamy, and *h*<1 to polyandry (including polyandrogyny in the sense of Shuster and Wade [27]). Formula (3) reduces to the frequently used minimum function *p*(*m*,*f*) = min(*hm*/*f*,1) in the absence of the mate-finding Allee effect (*θ*→0) and to (2) if the constraints on male mating potential are removed (*h*→∞); see [18] and [28] for details.

**Table 1:** Summary of the dynamics of the predator-prey system (4).

		female-biased predation	male-biased predation
unlimited polygyny	mating function (2)	I: extinction	II: coexistence possible (stable equilibrium)
limited polygyny	mating function (3) with 1<*h*<∞	I: cycles or extinction	II: coexistence possible (stable equilibrium or cycles)
polyandry	mating function (3) with *h*<1	III: coexistence possible (stable equilibrium)	IV: coexistence possible but very unlikely (stable equilibrium or cycles)

Different types of sex-selective predation in columns and different prey mating systems in rows. Roman numerals correspond to the areas in Fig. 4B. Extinction includes increasing oscillations that drop very close to zero.

To reduce the number of parameters, we scale all population densities in model (1) by a multiplicative factor *λ*_2_>0 and introduce predation bias Λ = *λ*_1_/*λ*_2_ (male bias: Λ>1, female bias: Λ<1) and a new Allee effect parameter Θ = *λ*_2_ *θ*:
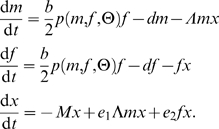
(4)For simplicity, we keep the same notation *m*, *f*, and *x* for the rescaled state variables as in model (1): whether we use model (1) or (4) is always clear from the context and the only difference in the rescaled mating functions (2) and (3) is that Θ replaces *θ*.

Inevitably, the dynamics and long-term stability of any predator-prey system will be affected by a multitude of various mechanisms, often with opposite impacts, and additional mechanisms may overshadow the effect of sex-selective predation. For example, negative density dependence in prey growth is known to have a strong stabilizing effect in predator-prey interactions [29]. We account for negative density dependence in prey growth and different types of predator-prey interactions (different forms of the functional and/or numerical response) in supplementary analyses (Text S2). To demonstrate their additional impact on the stability of the predator-prey equilibrium, we introduce them one by one in the basic model (4) with unlimited polygyny and no Allee effect.

Model (4) admits at most three steady states: the extinction equilibrium *E* ^0^, prey-only equilibrium *E* ^1^, and predator-prey equilibrium *E* ^2^ (Text S2). *E* ^1^ is unstable and *E* ^0^ locally stable if Θ>0. *E* ^1^ arises as a direct consequence of the mate-finding Allee effect in prey, and we call the prey density at *E* ^1^ the Allee threshold: a prey population above it will grow, but a decline to extinction occurs if the prey falls below. *E* ^0^ is unstable, i.e. both populations can recover from near-extinction, if there is no Allee effect (Θ = 0). We analyze model (4) numerically using MATLAB 7 (The MathWorks, Inc.) package MATCONT [30], focusing primarily on the stability of the predator-prey equilibrium *E* ^2^. Throughout the paper, the stability of system (4) is used synonymously with the stability of *E* ^2^.

The structure of model (4) becomes particularly simple when mating opportunities are unlimited (*p* = 1): the male prey influences the female prey only indirectly through apparent competition via the shared predator. For unlimited mating opportunities, unbiased predation (Λ = 1), and equal initial densities of the male and female prey, model (4) is identical to the classic Lotka-Volterra predator-prey model and results in cycles characteristic of many other predator-prey models. When we furthermore include a carrying capacity for the prey in this simplified model with unbiased predation (Text S2), we recover a model describing the feedback between hunting effort and population density of the hunted species under open-access exploitation [23].

## Results

### Patterns in quantitative data

Altogether we found 45 studies on 81 different pairs of predator and prey taxa ( = species level except some cases in which one taxon was identified at genus or family level), spanning both experimental and observational studies in the laboratory and in the field (Tables 2 and S1). Some of the studies involve several predator-prey pairs in which either the prey or predators are closely related; to remove some of the possible taxonomic bias, we report data for both predator-prey pairs and studies in Table 2. Many of these studies were also not primarily targeted at sex-selective predation; the currently available quantitative data are therefore rather heterogeneous.

**Table 2:** Overview of sex-selective predation in the literature.

Prey		Predator							
		mollusc	arachnidan	insect	fish	reptile	bird	mammal	*all predators*
crustacean	studies	**1 (1m/-)**	-	**2 (1m/1f)**	**5 (3m/2f)**	**1 (-/1f)**	**2 (1m/1f)**	**1 (-/1f)**	**10 (6m/5f)**
	PP pairs	1 (1m/-)	-	2 (1m/1f)	6 (4m^*^/2f)	1 (-/1f)	7 (1m/6f)	1 (-/1f)	18 (7m^*^/11f)
arachnidan	studies	-	**1 (1m/-)**	-	-	-	-	-	**1 (1m/-)**
	PP pairs	-	1 (1m/-)	-	-	-	-	-	1 (1m/-)
insect	studies	-	**7 (6m/2f)**	**4 (3m/1f)**	-	-	**2 (-/2f)**	**2 (2m/-)**	**14 (10m/5f)**
	PP pairs	-	21 (19m^*^/2f)	12 (11m^*^/1f)	-	-	4 (-/4f)	5 (5m^*^/-)	42 (35m^*^/7f)
fish	studies	-	-	-	-	**1 (1m/-)**	**2 (-/2f)**	**1 (1m/-)**	**4 (2m/2f)**
	PP pairs	-	-	-	-	1 (1m^*^/-)	2 (-/2f^*^)	1 (1m/-)	4 (2m^*^/2f^*^)
amphibian	studies	-	-	-	-	**1 (1m/-)**	-	**1 (1m/-)**	**2 (2m/-)**
	PP pairs	-	-	-	-	1 (1m/-)	-	1 (1m/-)	2 (2m/-)
bird	studies	-	-	-	-	-	**3 (1m/2f)**	-	**3 (1m/2f)**
	PP pairs	-	-	-	-	-	3 (1m/2f)	-	3 (1m/2f)
mammal	studies	-	-	-	-	-	**3 (3m/1f)**	**9 (7m/2f)**	**11 (9m/3f)**
	PP pairs	-	-	-	-	-	5 (4m/1f)	6 (5m/2f^*^)	11 (9m/3f^*^)
*all prey*	studies	**1 (1m/-)**	**8 (7m/2f)**	**6 (4m/2f)**	**5 (3m/2f)**	**3 (2m/1f)**	**12 (5m/8f)**	**14 (11m/3f)**	**45 (32m/19f)**
	PP pairs	1 (1m/-)	22 (20m^*^/2f)	14 (12m^*^/2f)	6 (4m^*^/2f)	3 (2m^*^/1f)	21 (6m/15f^*^)	14 (12m^*^/3f^*^)	81 (57m^*^/25f^*^)

Number of studies reporting sex-selective predation (bold) and the number of predator-prey (abbreviated as PP) pairs of taxa investigated in major animal groups; m = reported male bias, f = reported female bias. Predator-prey pairs = usually species; in a few cases predators or prey given as genera or families (indicated by asterisk). Both male and female bias has been reported in some predator-prey pairs and studies; total number of studies or predator-prey pairs in a cell may be thus lower than the sum of male- and female-biased data following in the parentheses. Some studies included predators or prey from several major groups, and some predator or prey species were, in one or several studies, in pairs with species from several major groups; data in rows and columns do not sum up in such cases. All available data are included, among them studies with <10 prey individuals and statistically non-significant results.

Despite obvious gaps, data in Table 2 agree with the well-established notion of generally higher predation risk for males [3], [5]. Nineteen studies reported female-biased predation for only 25 predator-prey pairs, while males were identified as the more vulnerable sex in 32 studies and 57 predator-prey pairs (studies and predator-prey pairs with both male- and female-biased predation are included in both categories). The prevalence of male-biased predation is significant when both the number of studies (one-tailed binomial test, *n* = 51, *P* = 0.046) and the number of taxa pairs (one-tailed binomial test, *n* = 82, *P* = 0.0003) are considered. The prevalence of male bias is even higher when only studies with significant male or female bias in predation (one-tailed binomial test, *P*<0.05) and at least 10 consumed prey are taken into account, i.e. nine studies and 14 predator-prey pairs with female bias and 24 studies and 46 predator-prey pairs with male bias (one-tailed binomial tests of prevalence of male bias in studies: *n* = 33, *P* = 0.007; prevalence in taxa pairs: *n* = 60, *P*<0.0001).

Since detailed data on mating systems, predation mechanisms, extent and type of sexual dimorphisms, intensity of sexual selection and stationarity (or lack thereof) of predation bias are not readily available for most of the predators and prey, we aggregate both prey and predator species into several broad taxa groups (crustaceans, insects, fish, amphibians and reptiles, birds, and mammals) to provide preliminary insights. This breakdown suggests several major patterns for both predator-prey pairs and studies. High predation risk is widespread especially in systems with insect and mammal prey and with arachnidan and mammal predators. Female-biased predation is common only in systems with crustacean prey and birds feeding on invertebrates and fish (Table 2). We return to possible explanations of these patterns in the discussion.

Only a few studies measured the actual ratio of predation rates on male and female prey e.g., [24], [25] which corresponds directly to a predation-bias parameter Λ used in our model below. In all other cases we estimate Λ as the ratio between the observed numbers of male and female prey killed by the predator(s) during the study period (male bias in predation: Λ>1; female bias: Λ<1; no sex bias: Λ = 1). To correct Λ for prey sex ratio, we divide Λ by the actual male to female ratio in the standing prey population whenever this information is known and otherwise assume 1∶1 sex ratio (Text S1 and Table S1). This assumption might affect quantitative results which we present below. However, all 14 pairs for which we had to assume 1∶1 sex ratio in the prey involve strongly male-biased predation, which is probably of low intensity in most of these pairs. In such circumstances the balanced sex ratio can be maintained despite predation, and even moderate departures from it would still lead to only minor differences in the results (Text S1). In some cases, e.g. when only males are killed, the values of Λ exceed 100; we truncate them at Λ = 100. To focus on studies with a clear-cut evidence of sex-selective predation, we summarize only quantitative data on the 60 predator-prey pairs for which the male or female bias in predation was significant and which included at least 10 consumed prey.

**Figure 2:**
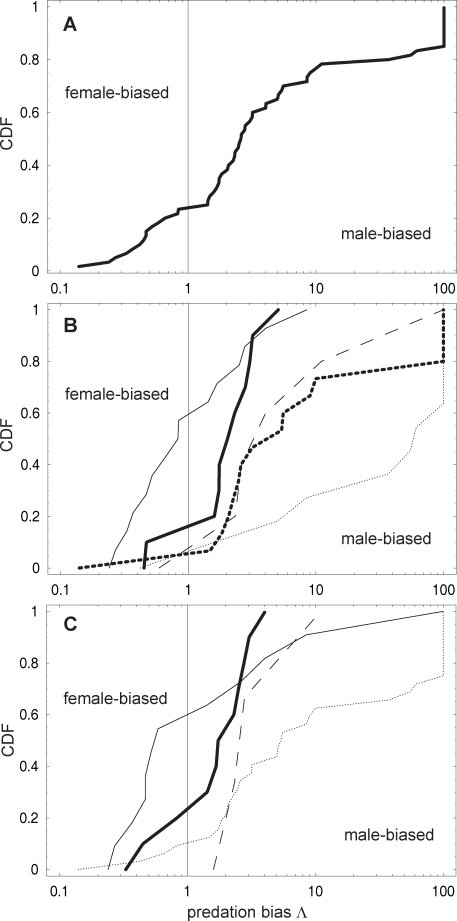
Cumulative distribution function (CDF) of empirical data on sex-biased predation. Λ = ratio of male/female prey eaten weighed by the prey sex ratio; large values truncated at Λ = 100. CDF(*z*) = fraction of predator-prey pairs with Λ≤*z*. A. All predator-prey pairs with significant results and at least 10 prey items (*n* = 60). B. Predator-prey pairs grouped over different predator taxa: thin dotted line = insects (*n* = 12); thick dashed line = arachnidans (*n* = 16); thin dashed line = molluscs, fishes and reptiles (*n* = 6); thin solid line = birds (*n* = 15); thick solid line = mammals (*n* = 11). C. Predator-prey pairs grouped over different prey taxa: thin dotted line = insects (*n* = 33); thin solid line = crustaceans (*n* = 12); thin dashed line = fishes and amphibians (*n* = 4); thick solid line = birds and mammals (*n* = 11).

The overall distribution of predation bias Λ shows that male-biased predation is not only more common but also reaches more extreme values (Fig. 2A). Eighteen predator-prey pairs were reported to have male-biased predation more extreme (Λ>7) than the most female-biased predation (Λ = 0.14 = 1/7). Data for predators (grouping all their prey together) suggest that strong male bias occurs mainly in insects (log_10_-transformed values of Λ, mean±1 S.D. = 1.37±0.83, *n* = 12), followed by arachnidans (0.81±0.82, *n* = 16), molluscs, fishes and reptiles grouped together (0.70±0.76, *n* = 6), and mammals (0.26±0.33, *n* = 11); while female bias is more common in bird predators (−0.02±0.46, *n* = 15, Fig. 2B). Insects also suffer the most male-biased predation as prey (0.93±0.85, *n* = 33, grouping all their predators together), followed by fishes and amphibians grouped together (0.52±0.37, *n* = 4), birds and mammals grouped together (0.18±0.35, *n* = 11), and crustaceans (0.11±0.77, *n* = 12; Fig. 2C). Differences in medians among these groups are significant both for predator and prey taxa (Kruskal-Wallis test; prey: *n* = 4, *P* = 0.003, predators: *n* = 5, *P* = 0.0003). Post-hoc pairwise comparisons (Dunn's test) revealed significant differences only between birds and insects (*P*<0.001) and birds and arachnidans (*P*<0.02) in predators, and between insects and crustaceans (*P*<0.01) and insects and birds+mammals (*P*<0.05) among prey groups.

### Model results

**Figure 3:**
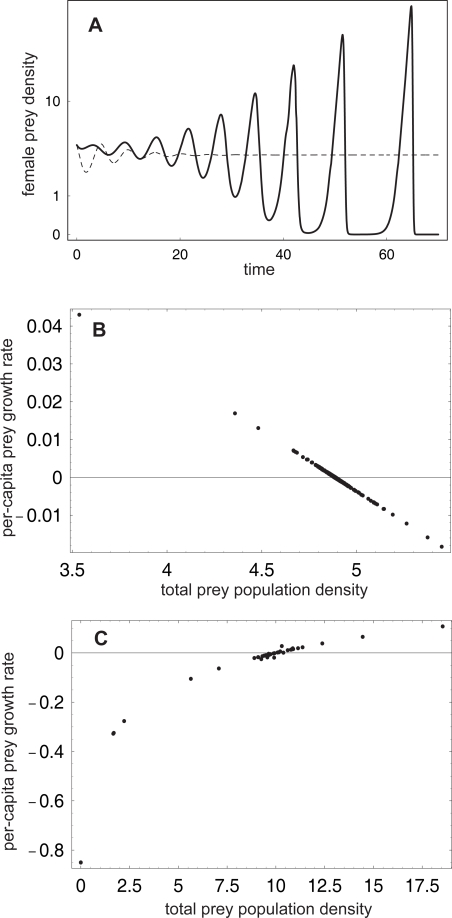
Illustration of population dynamics and the stabilizing and destabilizing effect of sex-selective predation in model (4). A. Two types of dynamics for unlimited polygyny and no Allee effect. Male-biased predation leads to a stable predator-prey equilibrium *E* ^2^ (thin dashed curve; Λ = 2); female-biased predation leads to increasing oscillations prone to collapse (thick curve; Λ = 0.5). Other parameters: *b* = 3, *d* = 0.2, Θ = 0, *e*_1_ = 0.2, *e*_2_ = 0.1, *M* = 1. Initial conditions: *m* = *f* = 4, *x* = 1.5. B. Stabilizing effect of the male-biased predation, shown in the per-capita population growth rate of the total prey population as a function of total prey density, *m*+*f*; data were generated by computing trajectories for ten random initial conditions and selecting points with predator density close to equilibrium, *x*∼*x* ^*^ (results for other fixed predator densities were similar). Λ = 2, other parameters as in A. C. Destabilizing effect of the female-biased predation, shown as in B. Λ = 0.5, other parameters as in A.

**Figure 4:**
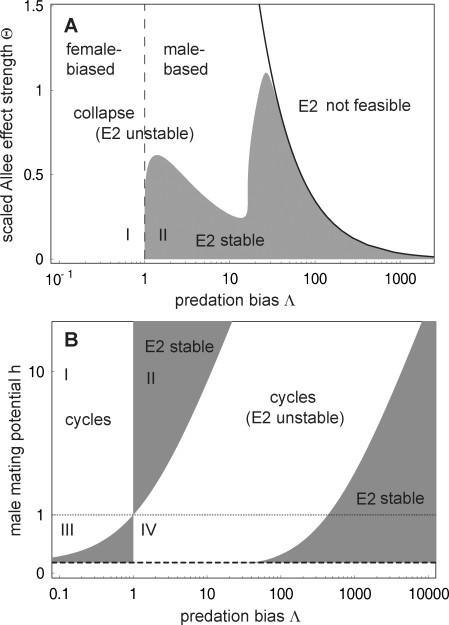
Stability of the predator-prey equilibrium *E* ^2^ in model (4). Common parameters: *b* = 3, *d* = 0.2, *e*_1_ = 0.2, *e*_2_ = 0.1, and *M* = 1. A. Combined effect of predation bias and the Allee effect under unlimited polygyny. *E* ^2^ is feasible to the left of the solid black curve and locally stable within the grey area. B. Combined effect of predation bias and prey mating system with no Allee effect (Θ = 0). The equilibrium is feasible above *h*∼0.133 (dashed line) and locally stable within each grey area. Areas I–IV delimited by lines *h* = 1 and Λ = 1 correspond to Table 1.

The stability of the predator-prey system (4) depends primarily on two factors: the prey mating system and predation bias for one sex of the prey. Male- and female-biased predation generally has opposite consequences for the stability (Table 1). The results are particularly simple for unlimited polygyny and no Allee effect: male-biased predation (Λ>1) leads to a stable coexistence, while female-biased predation (Λ<1) gives rise to increasing oscillations (Fig. 3A and Fig. 4A for Θ = 0).

The outcome for limited polygyny, i.e. finite *h*>1 in (3), is similar: only male-biased predation can lead to stable predator-prey equilibrium (area II in Fig. 4B and Table 1). In polyandrous prey (*h*<1), the roles of both sexes in prey dynamics are reversed, which is also reflected in the stabilizing role of sex-selective predation. Only female-biased predation or strongly male-biased predation can stabilize the predator-prey dynamics (areas III and IV in Fig. 4B and Table 1). Otherwise, sex-biased predation leads to stable predator-prey cycles (area I and parts of areas II, III and IV); often, the troughs of these cycles are very low and the system thus prone to collapse, e.g. due to the Allee effect in the prey (see below) or stochasticity.

To illustrate the mechanism causing the observed differences between male- and female-biased predation and different mating systems, we plot the per-capita growth rate of the entire prey population, 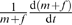, as a function of the total prey population density *m*+*f* (Fig. 3B and C). This illustration is not relevant for specialized predators that feed only on male or female prey (see Text S2 for analysis). Male-biased predation of polygynous prey gives rise to an emergent negative density-dependence in prey growth; populations perturbed away from the predator-prey equilibrium thus return to it (Fig. 3A and B). On the other hand, female-biased predation of polygynous prey leads to an emergent positive density dependence (i.e. not linked to the Allee effect if the latter is also present; see below) and thus has a destabilizing effect: predators feeding on female prey close to the equilibrium density first increase in numbers, while the female prey density decreases, leading to poor prey growth and subsequent die-off of the predators. As predators become scarce, the prey is released from predation and its density increases above the equilibrium level, followed by predators – these cycles spiral away from the predator-prey equilibrium *E* ^2^ (Fig. 3A and C). The (de)stabilizing effect of sex-biased predation is caused by the concomitant changes in male prey density: model (4) with male prey density kept fixed at an arbitrary value, no Allee effect and unlimited polygyny is a neutrally stable Lotka-Volterra predator-prey system.

**Figure 5:**
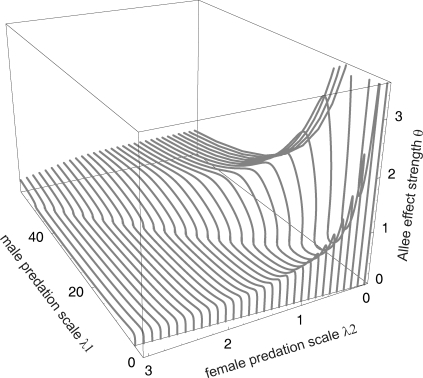
Combined effect of predation rates and the Allee effect in the prey in unscaled model (1). The curves trace a surface separating stable (below) and unstable (above) dynamics; points with *λ*_1_ = *λ*_2_ (shown for θ = 0, thin line bottom front) separate male- and female-biased predation. Other parameters as in Fig. 4A.

These conclusions do not change substantially in the presence of the mate-finding Allee effect (Θ>0). All additional differences in the results can be attributed to the presence of the Allee threshold. The prey population will fall below it and the predator-prey system can also collapse for male-biased predation (Λ>1). In terms of the unscaled model (1), the maximum strength *θ* of the mate-finding Allee effect allowing for stable predator-prey coexistence levels off asymptotically at highly male-biased predation for unlimited polygyny (Fig. 5). Such prey populations with a pronounced mate-finding Allee effect (high *θ*) can be stabilized only by predators that feed very little on females (low *λ*_2_) and moderately on males (intermediate *λ*_1_). The stability for limited polygyny and polyandry is limited in a similar way (Text S2 and Fig. S1). For all mating systems with the Allee effect, coexistence also becomes more difficult to achieve as predation strength relative to the intrinsic per-capita growth rate of the prey increases, e.g. through increased prey conversion efficiency *e*_i_ which leads to higher predator and lower prey density at the equilibrium (results not shown).

In the final set of results, we summarize the impact of various additional mechanisms on the dynamics. A finite prey carrying capacity stabilizes the dynamics, and stable coexistence becomes possible also for female-biased predation. The range of carrying capacities leading to stabilization can change with sex bias in predation (Text S2 and Fig. S2). A similar effect is observed when the predators are allowed to switch between the male and female prey to maximize their food intake rate (Text S2 and Fig. S3). On the contrary, a Holling type II functional response destabilizes the dynamics: as the handling time of the captured prey increases, the predator-prey equilibrium becomes unstable also for male-biased predation, which is stabilizing for the linear functional response, and the predation always leads to unstable dynamics above a certain critical handling time (Text S2 and Fig. S4).

## Discussion

Sex-selective predation can have important consequences for prey species with sexually dimorphic life histories. Mortality costs associated with sex-selective predation are a major force in the evolution of prey mating systems and sexual signalling [3] and the evolution of sexual size dimorphism [31]. However, little is known about their impact on the persistence and stability of predator-prey systems.

### Sex-selective predation and harvesting: population-dynamical consequences

We have shown, using a simple model of a predator feeding on sexually reproducing prey, that sex-selective predation should be taken into account along with other, well-established factors influencing the stability of predator-prey interactions. In the simplest setting, males affect females only indirectly through apparent competition via the shared predator. Males can also affect females directly via the mate-finding Allee effect. We demonstrated that the impact of sex-selective predation depends on the interplay of the predation bias and the prey mating system. Only predation on the ‘less limiting’ prey sex usually yields stable equilibria. This contrasts with predation on the ‘more limiting’ prey sex, which usually promotes unstable dynamics and thus makes the predator-prey system prone to collapse. Male-biased predation is therefore stabilizing in polygynous prey, while female-biased predation can only stabilize the dynamics if the prey mating system is polyandrous (Table 1). The presence of the Allee effect in the prey, apart from the collapse of the predator-prey system if the Allee effect is too strong, does not substantially alter these differences.

These results have general repercussions for predator-prey dynamics: many of the prey with quantified male-biased predation are likely to be polygynous (Table S1). For this class of prey, male-biased predation can stabilize the dynamics even if no other stabilizing mechanisms were present. The results are also puzzling: none of the prey with quantified female-biased predation is known to be polyandrous or polyandrogynous (Table S1). In general, polyandry/polyandrogyny is uncommon. How can female-biased predation exist? A value of our model lies in showing, among other things, that other stabilizing mechanisms, such as a finite carrying capacity of the prey or predator switching, can be essential for long-term coexistence of these predator-prey systems (Text S2 and Figs. S2 and S3). In intuitive terms, the negative density dependence in per-capita prey growth rate arising from such mechanisms must override the emergent positive density dependence brought by the female-biased predation. On the other hand, we demonstrate that the destabilization of the predator-prey dynamics by sex-selective predation can be further exacerbated, and stabilization overshadowed, by other mechanisms such as type II predator functional responses (Text S2 and Fig. S4).

Additional mechanisms can regulate systems with destabilizing sex-selective predation and prevent their extinction. For example, predator densities might be limited by some other (external) factors. Most predator-prey pairs are also embedded in larger food webs, and sex biases may on average cancel out if multiple predators share a prey as in the gypsy moth *Lymantria dispar* [32]–[34] or *Microtus* voles [35]. In that case, our simple model cannot give accurate predictions, although it might provide useful initial insight when the food web links between the prey and one of its predators are particularly strong; strong links with sex-selective predation have been reported, e.g., between the predatory phytoseiid mite *Typhlodromus occidentalis* feeding on the herbivorous spider mite *Panonychus ulmi* [36], predatory bivalves feeding on males of harpacticoid copepods [37], and sparid fish *Lithognathus lithognathus* eating mostly males of the amphipod *Grandidierella lignorum* [38].

Bias towards one sex is also common to harvesting of commercially important species and trophy hunting. Our model can, along with predator-prey dynamics, describe the temporal dynamics in harvesting/hunting effort and the density of a harvested/hunted population subject to open-access exploitation [45]. Harvesting is usually male-biased in ungulates [13] and their mating systems are more or less polygynous; our model therefore predicts that moderate open-access exploitation tends to have a stabilizing effect. On the other hand, exploitation of many fish stocks is biased towards larger or more active individuals and may be therefore female- or male-biased depending on the species and type of gear [12], [46]. Over longer timescales, bias towards either sex might therefore contribute to stability or large fluctuations and collapses in open-access fisheries. We emphasize that our conclusions are only relative and focus only on the differences between male- and female-biased exploitation. Sustainability of any exploitation scheme and its impact on the target population should be assessed on a case-by-case basis, as it will be influenced by a number of other factors, among them the exploitation intensity, mating system and any Allee effects in the exploited population.

In sum, our model demonstrates that sex-selective predation can substantially affect predator-prey dynamics. However, current empirical evidence of that phenomenon is very limited. More data are clearly needed to rigorously scrutinize this mechanism and support additional modelling attempts and/or experiments. In what follows, we discuss in more detail the quantitative data we collected, review some of the proximate mechanisms involved in sex-selective predation and harvesting, and highlight the limitations that currently hamper a more direct link between any modelling attempts and the data.

### Sex-selective predation: data and mechanisms

Published quantitative data on sex-selective predation suggest that, overall, male bias in predation occurs about two times as frequently as female bias. This is in line with previous reports of mostly male-biased predation [3]. A number of proximate mechanisms, usually involving sexual selection in the prey, has been proposed and identified to explain the skew towards male-biased predation. However, the choice of taxa could have been non-random, leading to fewer studies showing female bias in predation; prevailing reports of male bias might stem from the keen interest of researchers in some topics of sexual selection such as mortality costs associated with mating. More studies targeting situations in which female bias is plausible are needed to verify that it is indeed uncommon.

Biases towards male prey also seem to be more extreme than towards female prey, and they differ among major taxonomic groups. Male bias is most pronounced in insects, both as predators and as prey. Several studies highlighted predators with extremely male-biased predation (Λ>100) stemming from active exploitation of prey mating signals: bolas spiders producing ‘fake’ female sex pheromones of certain moth species [39], [40], tropical predatory fireflies mimicking female bioluminescence patterns of prey firefly species [41], and certain marine carnivorous bivalves feeding on copepods, in which the exact mechanism remains unknown [37].

Female bias is most often found in crustaceans among prey and in birds among predators. Reasons for it are more varied, although they may include sexual selection if the sexes play reversed roles in mating [42]. In crustaceans, females appear to be more conspicuous and/or less apt at escaping the predators than males [9], [10]; most birds are visual predators and their prey encounter rates will be enhanced by prey conspicuousness. Interestingly, predators which would specialize on prey mating signals and predate on females have not been reported. Potential prey of such predators includes, e.g. most butterflies and moths whose females produce sexual attractants [43]. It is possible that the tiny amounts of highly specific attractants are evolutionary adaptations of the females to predation risk, given that predators are probably under much lower selection pressure than the conspecific males to find the signalling females scattered in space [6].

### Linking the model and data

It is difficult, for at least three reasons, to quantitatively link the published quantitative data on sex-selective predation to model (4) and its extensions including other mechanisms influencing its stability. We have already mentioned one of the reasons: most predator-prey pairs are embedded in larger food webs and their population dynamics are affected by interactions with other species. Harvesting and trophy hunting is often at least partially regulated or, on the other hand, leads to a quick depletion of the exploited species; the (de)stabilizing effect of sex-selective predation probably makes a minor contribution to the long-term stability of such systems. Second, many of the observations were limited in space and time. Predation pressure on male and female individuals can vary over their lifetime e.g., [47], [33] and in different locations e.g., [48], [49]. Unfortunately, data on how predators might adjust their diet with respect to changes of relative male prey and female prey densities are currently missing.

Third, our analysis re-emphasizes that the mate-finding Allee effect destabilizes simple predator-prey systems and can lead to extinction of both predator and prey populations [20], [22]. However, the presence and strength of the mate-finding Allee effect and sometimes even the mating system are unknown for all prey species listed in Table S1 except the gypsy moth. Mating success in this species corresponds well to mating function (2) associated with unlimited polygyny [50], and leads to bistable population dynamics [51]. However, several predators with different prey sex selectivity interact with the gypsy moth, preventing us from the possibility to fit the model to these data.

### The evolutionary dimension of sex-selective predation

Why does sex-selective predation exist at all and which underlying (co)evolutionary processes lead to it? Explanations of sex-selective predation listed above are largely supported by mechanisms focusing on individual life history of the prey. That is, sex-specific predation always reveals some kind of sexual dimorphism in the prey that arises, e.g., from sexual selection and is only subsequently exploited by a predator. Although the bias (or the lack thereof) in predation will depend on the nature of the dimorphism and predator's foraging ecology, one might speculate that some components of sexual dimorphism are easier to exploit by predators and therefore limit the variation between sex bias in predation and the prey mating system. For example, polygyny often implies more conspicuous males and may thus lead to male-biased predation, while females are more conspicuous in polyandrous species and thus more likely to be preyed upon. The biased predation can also feed back to the sexual dimorphism of the prey, and lead to coevolutionary dynamics between the prey and predators; their exploration is beyond the limits of this paper.

Finally, we combine an evolutionary and population-dynamical argument to provide one more possible explanation of the observed skew towards male-biased predation. Given our theoretical results, it seems plausible that the skew reflects the evolutionary history of sex-selective predator-prey interactions. The inherent instability of female-biased predation might have prevented the persistence of such systems on longer timescales if other counter-acting stabilizing mechanisms have been absent or weak, leading to population-level selection. Current evidence for this hypothesis is weak due to lack of direct evidence, which should simultaneously include time series of predator and prey densities, information on the sex bias in predation, the mating system, and the presence and strength of other mechanisms influencing prey stability. Data in Tables 2 and S1 provide only circumstantial evidence: with the exception of the seasonally specialized birds feeding on Antarctic krill [44], none of the reviewed predator-prey systems appears to involve a single predator specialized on a particular prey and feeding predominately on females.

### Concluding remarks

We believe that more focus on sex-selective predation can yield additional and interesting insights as to which mechanisms maintain the persistence of predator-prey pairs over ecological and evolutionary timescales. Based on our review of standing empirical evidence of sex-selective predation, web-building spiders might serve as good model predators in terrestrial ecosystems and copepods as a useful prey model in aquatic environment. Predation biases found in these two groups are opposite, as copepod females are eaten more than males while spiders capture considerably more male than female prey. The combination of sex-selective predation and narrow spectra of prey is even more common in parasitoids, in which the impact of sex-selective parasitism is similar to predation (Berec and Boukal, unpublished work). To extend our study, it would be interesting to use the magnitude of sexual dimorphism or the intensity of sexual selection in the focal prey species as more detailed, quantitative predictors of sex bias in predation, given that the bias depends on the interaction of predator's behaviour and the type of sexual dimorphism in the prey. Our results also have implications for population dynamics of sexually dimorphic species with unequal exploitation of males and females. Our expectations are that under open access, harvesting and trophy hunting biased towards the ‘less limiting’ sex (usually males) should be more sustainable than a bias towards the other sex. These expectations can be verified by comparing the long-term stability of exploitation in a range of sexually dimorphic species with a different bias in exploitation.

## Supporting Information

Table S1 Table summarizing all published quantitative data on sex-selective predation.
(0.07 MB XLS)

Text S1 References and comments on published quantitative data on sex-selective predation in Table S1.
(0.07 MB DOC)

Text S2 Additional results and extensions of model (4). Here we examine the impact of mate-finding Allee effect on the predator-prey dynamics described by model (4) for prey with mating systems corresponding to limited polygyny and polyandry. We also outline how the main results of the paper change when other mechanisms affect stability of the predator-prey equilibrium together with sex-selective predation.
(0.10 MB DOC)

Figure S1 Stability of model (S1) in Text S2 with various mating systems and the mate-finding Allee effect. Precise extent of parameter combinations leading to stable cycles not shown. Common parameters: *b* = 3, *d* = 0.2, *e*_1_ = 0.2, *e*_2_ = 0.1, and *M* = 1. A. Combined effect of predation bias and prey mating system with a mate-finding Allee effect (Θ = 0.2). *E* ^2^ is feasible approximately above *h* = 0.133 and below Λ = 200 (thick solid line) and locally stable within each grey area. Areas I–IV delimited by lines *h* = 1 and Λ = 1 refer to Table 2 in the main text. B. Combined effect of predation bias and the Allee effect for limited polygyny (*h* = 3), except the dotted curve that delimits the area of stable *E* ^2^ for unlimited polygyny (infinite *h*).
(1.02 MB TIF)

Figure S2 Stability of model (S1) in Text S2 with unlimited polygyny and no mate-finding Allee effect. Combined effect of predation bias and parameter *K* scaling the prey carrying capacity. Other parameters: *b* = 3, *d* = 0.2, Θ = 0, *e*_1_ = 0.2, *e*_2_ = 0.1, and *M* = 1. *E* ^2^ is locally stable within the grey area. Areas I and II delimited by line Λ = 1 refer to Table 2 in the main text.
(0.95 MB TIF)

Figure S3 Stability of model (S2) in Text S2 with unlimited polygyny and no mate-finding Allee effect. Combined effect of predation bias and steepness in predator switching on the stability of the predator-prey equilibrium *E* ^2^ of model (S2). Parameters: *b* = 3, *d* = 0.2, Q = 0, *e*_1_ = 0.2, *e*_2_ = 0.1, and *M* = 1. *E* ^2^ is locally stable within the grey area. Areas I and II delimited by line Λ = 1 refer to Table 2 in the main text.
(0.96 MB TIF)

Figure S4 Stability of model (S3) in Text S2 with unlimited polygyny and no mate-finding Allee effect. Combined effect of predation bias and handling time of the predator with Holling type II functional response. Other parameters: *b* = 3, *d* = 0.2, Θ = 0, *e*_1_ = 0.2, *e*_2_ = 0.1, and *M* = 1. *E* ^2^ is locally stable within the grey area. Areas I and II delimited by line Λ = 1 refer to Table 2 in the main text.
(0.93 MB TIF)
