# Supplementary material for: Does Sex-Selective Predation Stabilize or Destabilize Predator-Prey Dynamics?
Source: PLoS One. 2008 Jul 16;3(7):e2687. doi: 10.1371/journal.pone.0002687 (PMC2444021; doi:10.1371/journal.pone.0002687)
Supplement: Text S1 — References and comments on published quantitative data on sex-selective predation in Table S1. (0.07 MB DOC) [file pone.0002687.s002.doc]

**Does Sex-Selective Predation Stabilize or Destabilize Predator-Prey Dynamics?**

David S. Boukal, Luděk Berec, Vlastimil Křivan

**Text S1: References and comments on Table S1**

The Excel sheet contains all quantitative data on sex-selective predation that we found in the literature.

**Comments on entries**

*Λ estimate (see below)*: $\Lambda=\frac{m_{\text{eaten}}}{f_{\text{eaten}}}\frac{f_{0}}{m _{0}}.$

*Initial numbers of male and female prey, m*_0_ *and f*_0_:

- if both integers and one or both larger than 1: *f*_0_ and *m*_0_ given in the original paper as numbers of individuals;

- if both smaller or equal to 1: *f*_0_ and *m*_0_ given only as ratios.

*Data on eaten prey*, *m*_eaten_ *and f*_eaten_*:*

- if both integers: numbers of individuals;

- if bold and blue: percentages (numbers cannot be retrieved from the original paper).

*Prey sex ratio:*$p_{0}=\frac{m _{0}}{m _{0}+f_{0}}.$

*Test on bias (one-sided binomial exact test):*

- male: null hypothesis is *p*  *p*_0_;

- female: null hypothesis is *p*  *p*_0_.

Untested biases (due to insufficient data) in parentheses; non-significant results (*P* > 0.05) italicized on grey background.

NA = data not available.

**Estimation of prey sex bias in predation rates from data**

Most data in Table S1 report the number of male and female prey killed by the predator during a certain, sometimes unknown period (*t*, *t*+Δ*t*). Per-capita male and female prey mortality rates due to predation equal *λ*_1_*x* and *λ*_2_*x*, respectively. Assuming that the predator population *x* is constant during that period, *x*Δ*t* is small and natural mortality of the prey is negligible, the sex ratio of killed prey equals

$$\frac{m_{\text{eaten}}(\Delta t)}{f_{\text{eaten}}(\Delta t)}=\frac{m(t)-m(t+\Delta t)}{f(t)-f(t+\Delta t)}=\frac{m(t)\left(1-\text{exp}(-\lambda_{ 1}x\Delta t)\right)}{f(t)\left(1-\text{exp}(-\lambda_{ 2}x\Delta t)\right)}\sim\mu\frac{\lambda_{ 1}x\Delta t}{\lambda_{ 2}x\Delta t},$$

where *μ* is the male-to-female prey ratio at time *t*. As a result,

$$\Lambda=\frac{\lambda_{ 1}}{\lambda_{ 2}}\sim\frac{1}{\mu}\frac{m_{\text{eaten}}(\Delta t)}{f_{\text{eaten}}(\Delta t)},$$

which we use for estimating Λ in data unless the rates *λ*_1_ and *λ*_2_ have been measured directly.

Two potential sources of error can enter this estimate. First, some of the field estimates of sex ratios can be biased by population census methods that record sexes disproportionately (e.g., Acharya 1995). Second, the approximation will be incorrect for high predation pressure *x*Δ*t* (long observation period and/or many predators); however, predation will still be classified correctly as male- or female-biased if the prey sex ratio estimate is good.

**Sensitivity of results on the 1:1 prey sex ratio assumption for species with no information**

We had to assume 1:1 prey sex ratio for 14 predator-prey pairs with male-biased predation (*Λ* > 2.6) in Table S1: one crustacean and 13 insect prey, and one fish, four arachnidan, and nine insect predators (Burk 1982, Yeargan 1988, Nakagami et al. 2000). To assess the sensitivity of the predation bias on these data, we carried out two supplementary analyses with the prey sex ratio increased and decreased by 50% (i.e., 1.5:1 and 1:1.5) in all 14 pairs. In eight pairs, only males were preyed upon, and the data remained unchanged in the sensitivity analysis. The adjusted log_10_-transformed values of *Λ* are summarized in the following table (Table S2); the results of the Kruskal-Wallis tests on significance and post-hoc comparisons were qualitatively identical to those based on the 1:1 prey sex ratio assumption in the main text:

| **Sex ratio (*m*_0_:*f*_0_)** | 1:1 | 1.5:1 | 1:1.5 |
| --- | --- | --- | --- |
| **Predators** |  |  |  |
| insects (*n*=12) | 1.37 ± 0.83 | 1.32 ± 0.83 | 1.43 ± 0.84 |
| arachnidans (*n*=16) | 0.81 ± 0.82 | same | same |
| molluscs+fishes+ reptiles (*n*=6) | 0.70 ± 0.76 | 0.67 ± 0.77 | 0.73 ± 0.75 |
| mammals (*n*=11) | 0.26 ± 0.33 | same | same |
| birds (*n*=15) | -0.02 ± 0.46 | same | same |
|  |  |  |  |
| **Prey** |  |  |  |
| insects (*n*=33) | 0.93 ± 0.85 | 0.91 ± 0.84 | 0.96 ± 0.86 |
| fishes+ amphibians (*n*=4) | 0.52 ± 0.37 | same | same |
| mammals+ birds (*n*=11) | 0.18 ± 0.35 | same | same |
| crustaceans (*n*=12) | 0.11 ± 0.77 | 0.09 ± 0.77 | 0.12 ± 0.78 |

**Table S2:** **Sensitivity of results on the 1:1 prey sex ratio assumption for species with no information.** Data represent log_10_-transformed values of Λ (mean ± 1 S.D.) for predators (grouping all their prey together) and prey (grouping all their predators together). Same = data unchanged.

**References**

Acharya L (1995) Sex-biased predation on moths by insectivorous bats. Animal Behaviour 49: 1461–1468.

Blais JM, Maly EJ (1993) Differential predation by *Chaoborus americanus* on males and females of two species of *Diaptomus*. Canadian Journal of Fisheries and Aquatic Sciences 50: 410–425.

Britton RH, Moser ME (1982) Size specific predation by herons and its effect on the sex-ratio of natural populations of the mosquito fish *Gambusia affinis*. Oecologia 53: 146–151.

Burk T (1982) Evolutionary significance of predation on sexually signalling males. Florida Entomologist 65: 90–104.

FitzGibbon CD (1990) Why do hunting cheetahs prefer male gazelles. Animal Behaviour 40: 837–845.

Garcia CM, Saborio E, Berea C (1998) Does male-biased predation lead to male scarcity in viviparous fish? Journal of Fish Biology 53: 104–117, Suppl. A.

Götmark F, Post P, Olsson J, Himmelmann D (1997) Natural selection and sexual dimorphism: sex-biased sparrowhawk predation favours crypsis in female chaffinches. Oikos 80: 540–548.

Gwynne DT (1987) Sex-biased predation and the risky mate-locating behaviour of male tick-tock cicadas (Homoptera: Cicadidae). Animal Behaviour 35: 571–576.

Gwynne DT, Bussière LF (2002) Female mating swarms increase predation risk in a ‘role-reversed’ dance fly (Diptera: Empididae: *Rhamphomiya longicauda* Loew). Behaviour 139: 1425–1430.

Gwynne DT, O’Neill KM (1980) Territoriality in digger wasps results in sex biased predation on males (Hymenoptera: Sphecidae, *Philanthus*). Journal of the Kansas Entomological Society 53: 220–224.

Hairston NG, Walton WE, Li KT (1983) The causes and consequences of sex-specific mortality in a freshwater copepod. Limnology and Oceanography 28: 935–947.

Harrison S, Hebert D (1988) Selective predation by cougar within the Junction Wildlife Management Area. Biennial Symposium of the Northern Wild Sheep and Goat Council 6: 292–306.

Hendrichs MA, Hendrichs J (1998) Perfumed to be killed: interception of Mediterranean fruit fly (Diptera: Tephritidae) sexual signalling by predatory foraging wasps (Hymenoptera: Vespidae). Annals of the Entomological Society of America 91: 228–234.

Hicks GRF, Marshall BA (1985) Sex selective predation of deep-sea, meiobenthic copepods by pectinacean bivalves and its influence on copepod sex ratios. New Zealand Journal of Marine and Freshwater Research 19: 227–231.

Howard RD (1981) Sexual dimorphism in bullfrogs. Ecology 62: 303–310.

Johnson D, Proctor HC (1991) Effects of phytoseiid predators on the sex-ratio of the spider-mite *Panonychus ulmi*. Canadian Journal of Zoology 1991: 208–212.

Karban R (1983) Sexual selection, body size and sex-related mortality in the cicada *Magicicada cassini*. American Midland Naturalist 109: 324–330.

Kittlein MJ, Vassallo AI, Busch C (2001) Differential redation upon sex and age classes of tuco-tucos (*Ctenomys talarum*, Rodentia: Octodontidae). Mammalian Biology 66: 281–289.

Koga T, Backwell PRY, Christy JH, Murai M, Kasuya E (2001) Male-biased predation of a fiddler crab. Animal Behaviour 62: 201–207.

Koivunen V, Korpimäki E, Hakkarainen H (1996) Differential avian predation on sex and size classes of small mammals: doomed surplus or dominant individuals? Annales Zoologici Fennici 33: 293–301.

Krausman PR, Leopold BD, Seegmiller RF, Torres SG (1989) Relationships between desert bighorn sheep and habitat in Arizona. Wildlife Monographs 102.

Lodé T, Holveck MJ, Lesbarrères D, Pagano A (2004) Sex-biased predation by polecats influences the mating system of frogs. Proceedings of the Royal Society London B (Supplement) 271: S399–S401.

Maly EJ (1970) The influence of predation on the adult sex ratios of two copepod species. Limnology and Oceanography 15: 566–573.

Mœller AP, Nielsen JT (1997) Differential predation cost of a secondary sexual character: sparrowhawk predation on barn swallows. Animal Behaviour 54: 1545–1551.

Moore SD (1987) Male-biased mortality in the butterfly *Euphydryas editha*: a novel cost of mate acquisition. The American Naturalist 130: 306–309.

Mooring MS, Fitzpatrick TA, Nishihira TT, Reisig DD (2004) Vigilance, predation risk, and the Allee effect in desert bighorn sheep. Journal of Wildlife Management 68: 519–532.

Nakagami M, Takatsu T, Matsuda T, Takahashi T (2000) Feeding on harpacticoid copepods by marble sole *Pleuronectes yokohamae* juveniles in the coastal areas of Tsugaru Strait, Hokkaido. Nippon Suisan Gakkaishi 66: 818–824.

Norddahl K, Korpimäki E (1998) Does mobility or sex of voles affect risk of predation by mammalian predators? Ecology 79: 226–232.

Ohsaki N (1995) Preferential predation of female butterflies and the evolution of Batesian mimicry. Nature 378: 173–175.

Owen-Smith N (1993) Comparative mortality rates of male and female kudus: the costs of sexual dimorphism. Journal of Animal Ecology 62: 428–440.

Polis GA, Barnes JD, Seely MK, Henschel JR, Enders MM (1998) Predation as a major cost of reproduction in Namib desert tenebrionid beetles. Ecology 79: 2560–2566.

Post P, Götmark F (2006) Foraging behaviour and predation risk in male and female Eurasian blackbirds (*Turdus merula*) during the breeding season. The Auk 123: 162–170.

Quinn TP, Kinnison MT (1999) Size-selective and sex-selective predation by brown bears on sockeye salmon. Oecologia 121: 273–282.

Rehfeldt G (1992) Impact of predation by spiders on a territorial damselfly (Odonata, Calopterygidae). Oecologia 89: 550–556.

Reid K, Trathan PN, Croxall JP, Hill HJ (1996) Krill caught by predators and nets: differences between species and techniques. Marine Ecology Progress Series 140: 13–20.

Schaeffer RJ, Torres SG, Bleich VC (2000) Survivorship and cause-specific mortality in sympatric populations of mountain sheep and mule deer. California Fish and Game 86: 127–135.

Schlacher TA, Wooldridge TH (1996) Patterns of selective predation by juvenile, benthivorous fish on estuarine macrofauna. Marine Biology 125: 241–247.

Smith DW, Drummer TD, Murphy KM, Guernsey DS, Evans SB (2004) Winter prey selection and estimation of wolf kill rates in Yellowstone National Park, 1995-2000. Journal of Wildlife Management 68: 153–166.

Sommer S (2000) Sex-specific predation on a monogamous rat, *Hypogeomys antimena* (Muridae: Nesomyinae). Animal Behaviour 59: 1087–1094.

Svensson JE (1997) *Chaoborus* predation and sex-specific mortality in a copepod. Limnology and Oceanography 42: 572–577.

Svensson JE (1997b) Fish predation on *Eudiaptomus gracilis* in relation to clutch size, body size, and sex: a field experiment. Hydrobiologia 344: 155–161.

Thornhill R (1978) Some arthropod predators and parasites of adult scorpionflies (Mecoptera). Environmental Entomology 7: 714–716.

Trexler JC, Tempe RC, Travis J (1994) Size-selective predation of sailfin mollies by 2 species of heron. Oikos 69: 250–258.

Weseloh RM (1988) Prey preferences of *Calosoma sycophanta* L. (Coleoptera: Carabidae) larvae and relation of prey consumption to predator size. Canadian Entomologist 120: 873–880.

Yeargan KV (1988) Ecology of a bolas spider, *Mastophora hutchinsoni* – phenology, hunting tactics, and evidence for aggressive chemical mimicry. Oecologia 74: 524–530.
