# Supplementary material for: Does Sex-Selective Predation Stabilize or Destabilize Predator-Prey Dynamics?
Source: PLoS One. 2008 Jul 16;3(7):e2687. doi: 10.1371/journal.pone.0002687 (PMC2444021; doi:10.1371/journal.pone.0002687)
Supplement: Text S2 — Additional results and extensions of model (4). Here we examine the impact of mate-finding Allee effect on the predator-prey dynamics described by model (4) for prey with mating systems corresponding to limited polygyny and polyandry. We also outline how the main results of the paper change when other mechanisms affect stability of the predator-prey equilibrium together with sex-selective predation. (0.10 MB DOC) [file pone.0002687.s003.doc]

**Does Sex-Selective Predation Stabilize or Destabilize Predator-Prey Dynamics?**

David S. Boukal, Luděk Berec, Vlastimil Křivan

**Text S2: Additional results and extensions of model (4)**

This additional information describes the impact of mate-finding Allee effect on the predator-prey dynamics described by model (4) for prey with mating systems corresponding to limited polygyny and polyandry. We also outline how the main results presented in this paper change when other mechanisms affect stability of the predator-prey equilibrium together with sex-selective predation. We discuss the influence of logistic prey growth, numerical response of the predator (based on optimal foraging theory), and Holling type II functional response of the predator in combination with the prey mating system corresponding to unlimited polygyny and no mate-finding Allee effect. Finally, we summarize the outcome of the unscaled model (1) when predators feed only on one sex of the prey.

### Equilibria of model (4)

The rescaled model (4) admits at most three equilibria. Introducing functions

$$\begin{matrix} \Phi_{\Theta}(m)=p(m,m,\Theta), \\ \Psi_{\Theta,\Lambda}(m)=p\left(m,\frac{1}{e_{2}}\left(M-e_{1}\Lambda m\right),\Theta\right), \end{matrix}$$

(both of them increase from 0 to 1 as *m* grows from 0 to +∞) and assuming positivity of the second argument in Ψ_Θ,Λ_(*m*), the equilibria can be written as

$$E^{0}=\{0,0,0\} ,$$

$E^{1}=\left\{\bar{m},\bar{m},0\right\}=\left\{\Phi_{\Theta}^{-1}\left(\frac{2d}{b}\right),\Phi_{\Theta}^{-1}\left(\frac{2d}{b}\right),0\right\}$,

and

in which *m***^*^** is the (single) root of the equation

$$\frac{b}{2e_{2}}\left(M-\Lambda m(e_{1}+e_{2})\right)\Psi_{\Theta,\Lambda}(m)-dm(1-\Lambda)=0.$$

The origin *E*^0^ is always unstable in the absence of the Allee effect (when *θ *=0 and hence Θ=0) since we assume *b*>2*d*. In that case the prey-only equilibrium *E*^1^ disappears and the prey grows exponentially in the absence of predators. For the female mating rate functions *p* considered in this paper, *E*^0^ is locally stable and *E*^1^ unstable if the Allee effect is present (Θ>0). *E*^2^ is meaningful only if *m***^*^**, *f* **^*^** and *x***^*^** are all positive, and ceases to exist when it collides with *E*^1^ for Θ>0. We use the latter relationship to find parameter combinations for which *E*^2^ is feasible.

### The mate-finding Allee effect

In the main text we show that the mate-finding Allee effect limits the range of predation bias for which the predator-prey equilibrium can be stable under unlimited polygyny. Figure S1 illustrates the same effect for limited polygyny and polyandry. The Allee effect will take away cyclic predator-prey dynamics in which the prey densities fall too low, and stable predator-prey cycles which emerge for some parameter combinations (Fig. S1) thus have the prey density always bounded away from zero by the Allee threshold. Decreasing male mating potential in limited polygyny also has a destabilizing impact on the dynamics; for example, the range of the Allee effect and predation bias combinations leading to stable predator-prey coexistence is larger for unlimited than for limited polygyny (Fig. S1B; area below the dotted curve and grey areas, respectively).

### Logistic prey growth

We capture the logistic prey growth in model (4) by considering negative density dependence in prey mortality rate:

$\begin{matrix} \frac{\text{d}m}{\text{d}t}=\frac{b}{2}p(m,f,\Theta)f-d\left(1+\frac{m+f}{K}\right)m-\Lambda mx \\ \frac{\text{d}f}{\text{d}t}=\frac{b}{2}p(m,f,\Theta)f-d\left(1+\frac{m+f}{K}\right)f-fx \\ \frac{\text{d}x}{\text{d}t}=-Mx+e_{1}\Lambda mx+e_{2}fx . \end{matrix}$ (S1)

Modelled this way, prey survival rate decreases with total prey density. Carrying capacity of the prey in the absence of predation increases with *K* irrespective of the mating system. The stabilizing property of the logistic prey growth is shown in Fig. S2.

Model (S1) is based on rescaled variables and parameters, which also pertains to the carrying capacity of the prey: K=*λ*_2_*k*, where *k* would be the unscaled carrying capacity parameter in model (1). All else being equal, decreasing the predation bias Λ=*λ*_1_/ *λ*_2_ either corresponds to decreasing predation on male prey or increasing predation on female prey. As Λ decreases from 1 to 0 by lowering the predation on male prey (λ_1_), the destabilizing effect of female-biased predation, mediated indirectly by predation on male prey, weakens and allows for a wider range of carrying capacities to stabilize the dynamics. In the complete absence of predation on males (*λ*_1_=0), model (S1) turns into a Lotka-Volterra system with a carrying capacity in the prey, for which any finite carrying capacity stabilizes the dynamics. On the other hand, if the predation bias decreases from 1 to 0 through increased predation on female prey (*λ*_2_), the destabilizing effect of female-biased predation remains the same and the unscaled carrying capacity parameter *k* yielding a stable predator-prey equilibrium remains approximately constant (Fig. S2).

### Behavioural response of the predator (predator switching)

The predation bias for prey sex will no longer be constant if predators can adjust their foraging mode in response to changing male and female prey densities. One plausible mechanism involves optimal foraging, in which predators adjust their feeding to maximize their food intake rate [S1]. In this modification of model (4), we assume that predators use search images to locate the currently more profitable sex of the prey, i.e. they aim at maximizing their instantaneous food intake rate *e*_1_Λ*m*+*e*_2_*f*, and that the male and female prey search images are traded off against each other. We denote by *u* the probability that the predator will use a search image to capture only males. Optimal foraging theory predicts that predators with perfect information about their environment will eat only males (*u*=1) when *e*_1_Λ*m*>*e*_2_*f*  and only females (*u*=0) when *e*_1_Λ*m*<*e*_2_*f* . We embed this optimal foraging mode into a more general family of predator switching rules described by the function

$$u(m,f,a)=\frac{1}{1+\exp\left(-a(e_{1}\Lambda m-e_{2}f)\right)}.$$

In this formula, parameter *a* gives the slope of the switching: *a*→∞ corresponds to the optimal foraging mode described above, while *a*=0 gives *u*=0.5 irrespective of the male and female prey population densities.

We include this family of predator switching rules in the rescaled model (4) as:

$\begin{matrix} \frac{\text{d}m}{\text{d}t}=\frac{b}{2}p(m,f,\Theta)f-dm-2u(m,f,a)\Lambda mx \\ \frac{\text{d}f}{\text{d}t}=\frac{b}{2}p(m,f,\Theta)f-df-2\left(1-u(m,f,a)\right)fx \\ \frac{\text{d}x}{\text{d}t}=-Mx+2e_{1}u(m,f,a)\Lambda mx+2e_{2}\left(1-u(m,f,a)\right)fx . \end{matrix}$ (S2)

In this setting, *a*=0 represents model (4). The stabilizing property of the switching for unlimited polygyny (*h*→∞) and no mate-finding Allee effect (Θ=0) is illustrated in Fig. S3. The switching greatly enhances the stability of the predator-prey equilibrium *E*^2^ when predation is female-biased. Some combinations of male-biased predation and nearly optimal foraging give rise to a stable predator-prey limit cycle and hence destabilize *E*^2^. This predator-prey cycle with a limited amplitude, which is characteristic of optimal foraging [S2], can also arise for female-biased predation when the switching is sufficiently steep (high *a*).

This type of switching also corresponds to a food patch selection model of optimal foraging when the male and female prey are spatially segregated. Predators can either focus on the male or female patch. This case is probably not common but might occur for some ungulates such as one of the predator-prey pairs in our data set, the desert bighorn sheep *Ovis canadiensis* preyed upon by the mountain lion *Felis concolor* (Mooring et al. 2004).

### Holling type II functional response

Holling type II predator functional response is known to destabilize predator-prey dynamics [S3]. We include type II functional response in model (4), assuming the same handling time τ for male and female prey:

$\begin{matrix} \frac{\text{d}m}{\text{d}t}=\frac{b}{2}p(m,f,\Theta)f-dm-\frac{\Lambda mx}{1+\tau\Lambda m+\tau f} \\ \frac{\text{d}f}{\text{d}t}=\frac{b}{2}p(m,f,\Theta)f-df-\frac{fx}{1+\tau\Lambda m+\tau f} \\ \frac{\text{d}x}{\text{d}t}=-Mx+\frac{e_{1}\Lambda mx+e_{2}fx}{1+\tau\Lambda m+\tau f} . \end{matrix}$ (S3)

In this setting, τ=0 gives model (4). The increasingly destabilizing impact of the handling time is shown in Fig. S4.

### Predators feeding only on male or female prey

The rescaled model (4) does not cover specialized predators that feed on only one sex of the prey, and we return to the original unscaled model (1) with unlimited polygyny to explain the population consequences.

First, male prey will remain constant while female prey and predators will grow indefinitely if predators feed only on male prey and the mate-finding Allee effect is absent or limited in magnitude; strong Allee effects always lead to a collapse. Second, the dynamics of female prey and predator in model (1) reduce to the classical Lotka-Volterra predator-prey system with neutrally stable cycles surrounding the predator-prey equilibrium when predators feed only on female prey and there is no Allee effect. Any mate-finding Allee effect makes the equilibrium unstable and leads to collapse. The persistence of predator-prey systems with both types of specialized predation thus requires additional stabilizing mechanisms, such as the two discussed above.

**References**

S1 Stephens DW, Krebs JR (1986) Foraging theory. Princeton, NJ, USA: Princeton University Press.

S2 Křivan V (1997) Dynamic ideal free distribution: Effects of optimal patch choice on predator-prey dynamics. American Naturalist 149: 164–178.

S3 Murdoch WW, Oaten A (1975) Predation and population stability. Advances in Ecological Research 9: 1–131.

**Figure legends**

**Figure S1:** **Stability of model (S1) with various mating systems and the mate-finding Allee effect.**

Precise extent of parameter combinations leading to stable cycles not shown. Common parameters: *b*=3, *d*=0.2, *e*_1_=0.2, *e*_2_=0.1, and *M*=1. **A.** Combined effect of predation bias and prey mating system with a mate-finding Allee effect (Θ=0.2). *E*^2^ is feasible approximately above *h*~0.133 and below Λ~200 (thick solid line) and locally stable within each grey area. Areas I-IV delimited by lines *h*=1 and Λ=1 refer to Table 1 in the main text. **B.** Combined effect of predation bias and the Allee effect for limited polygyny (*h*=3), except the dotted curve that delimits the area of stable *E*^2^ for unlimited polygyny (*h*).

**Figure S2: Stability of model (S1) with unlimited polygyny and no mate-finding Allee effect.**

Combined effect of predation bias and parameter *K* scaling the prey carrying capacity. Other parameters: *b*=3, *d*=0.2, Θ=0, *e*_1_=0.2, *e*_2_=0.1, and *M*=1. *E*^2^ is locally stable within the grey area. Areas I and II delimited by line Λ=1 refer to Table 1 in the main text.

**Figure S3: Stability of model (S2) with unlimited polygyny and no mate-finding Allee effect.**

Combined effect of predation bias and steepness in predator switching on the stability of the predator-prey equilibrium *E*^2^ of model (S2). Parameters: *b*=3, *d*=0.2, Θ=0, *e*_1_=0.2, *e*_2_=0.1, and *M*=1. *E*^2^ is locally stable within the grey area. Areas I and II delimited by line Λ=1 refer to Table 1 in the main text.

**Figure S4: Stability of model (S3) with unlimited polygyny and no mate-finding Allee effect.**

Combined effect of predation bias and handling time of the predator with Holling type II functional response. Other parameters: *b*=3, *d*=0.2, Θ=0, *e*_1_=0.2, *e*_2_=0.1, and *M*=1. *E*^2^ is locally stable within the grey area. Areas I and II delimited by line Λ=1 refer to Table 1 in the main text.
