# Supplementary figures and images for: Does Sex-Selective Predation Stabilize or Destabilize Predator-Prey Dynamics?
Source: PLoS One. 2008 Jul 16;3(7):e2687. doi: 10.1371/journal.pone.0002687 (PMC2444021; doi:10.1371/journal.pone.0002687)

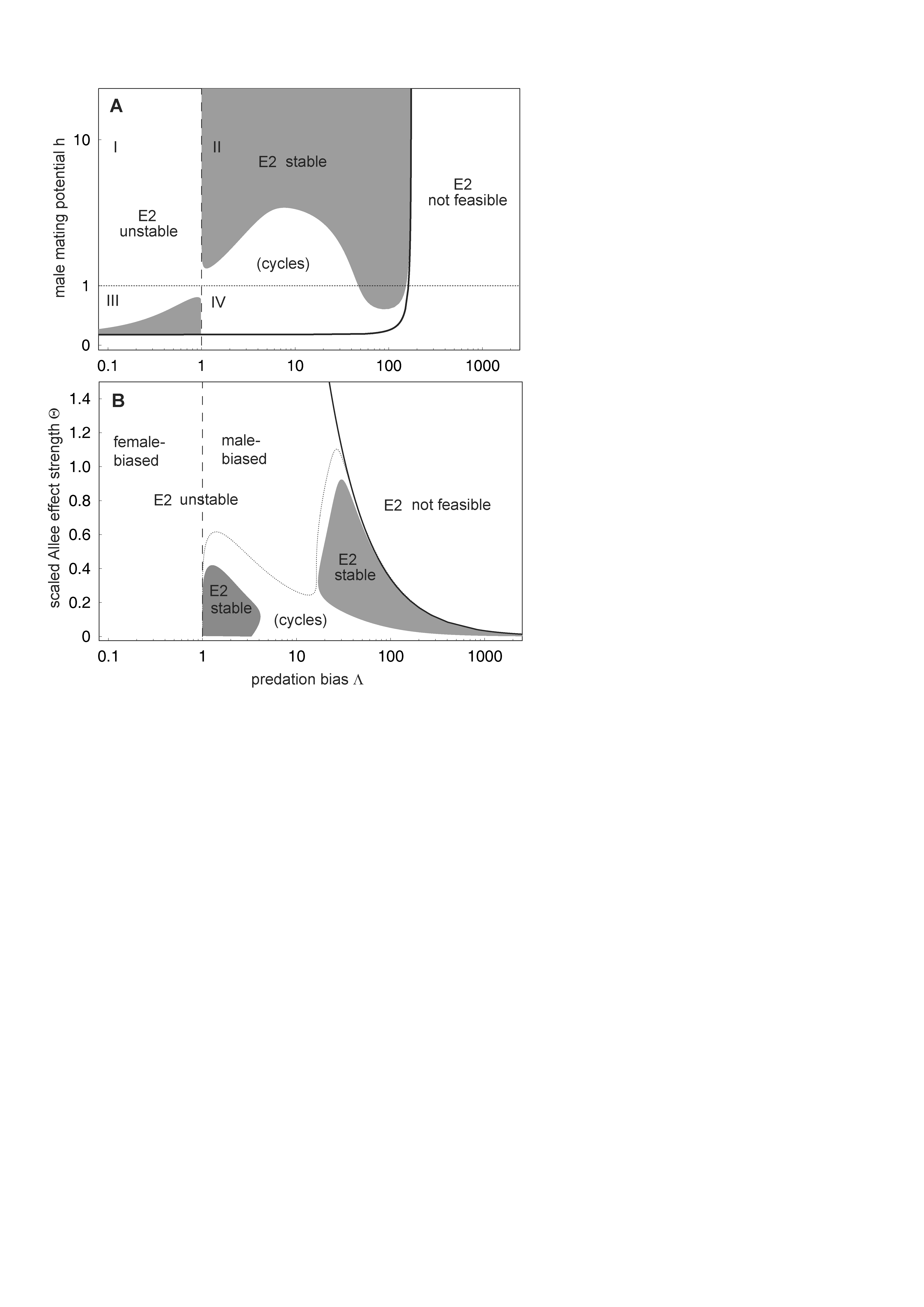

Supplement: Figure S1 — Stability of model (S1) in Text S2 with various mating systems and the mate-finding Allee effect. Precise extent of parameter combinations leading to stable cycles not shown. Common parameters: b = 3, d = 0.2, e 1 = 0.2, e 2 = 0.1, and M = 1. A. Combined effect of predation bias and prey mating system with a mate-finding Allee effect (Θ = 0.2). E 2 is feasible approximately above h = 0.133 and below Λ = 200 (thick solid line) and locally stable within each grey area. Areas I–IV delimited by lines h = 1 and Λ = 1 refer to Table 2 in the main text. B. Combined effect of predation bias and the Allee effect for limited polygyny (h = 3), except the dotted curve that delimits the area of stable E 2 for unlimited polygyny (infinite h). (1.02 MB TIF) [file pone.0002687.s004.tif]

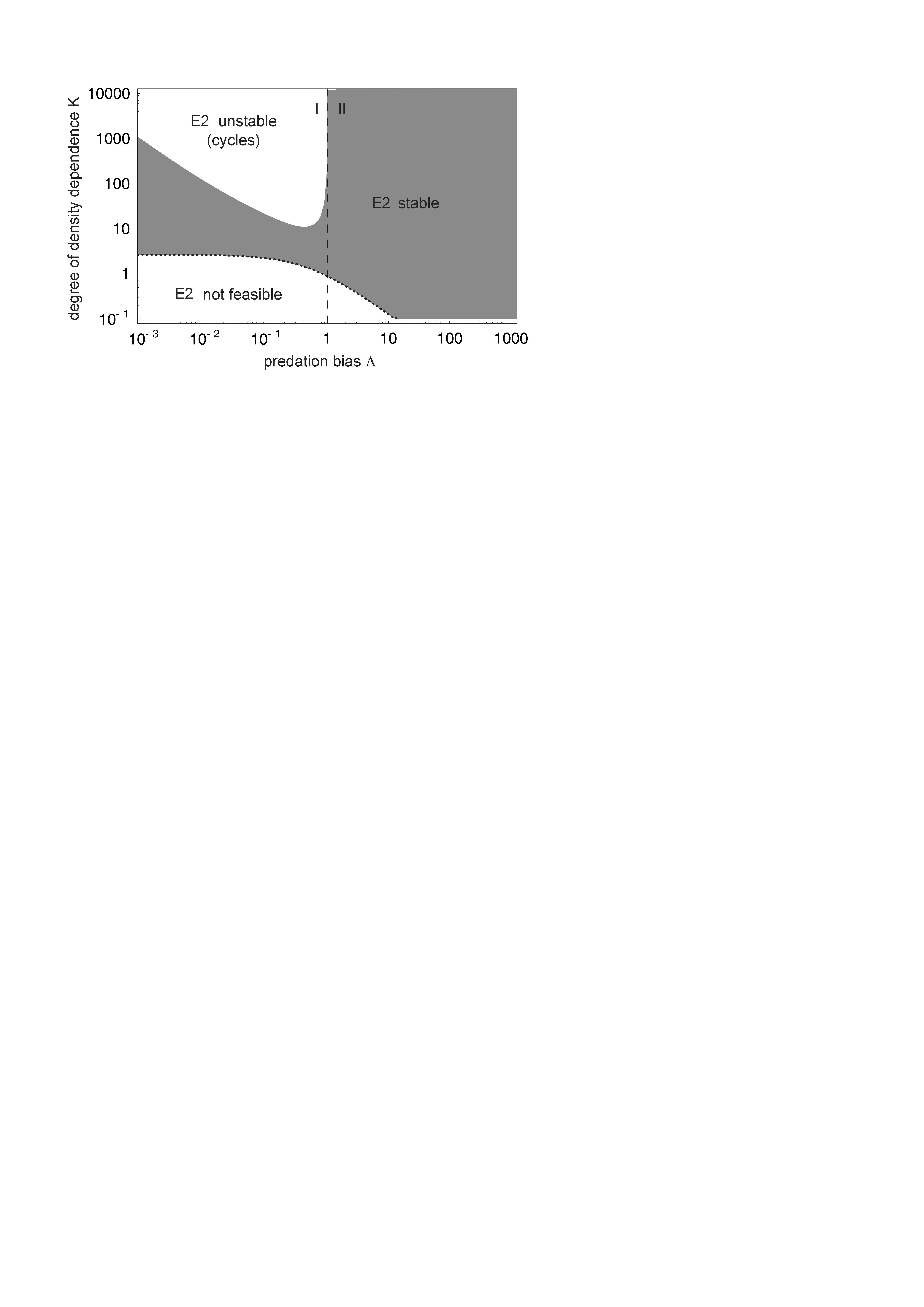

Supplement: Figure S2 — Stability of model (S1) in Text S2 with unlimited polygyny and no mate-finding Allee effect. Combined effect of predation bias and parameter K scaling the prey carrying capacity. Other parameters: b = 3, d = 0.2, Θ = 0, e 1 = 0.2, e 2 = 0.1, and M = 1. E 2 is locally stable within the grey area. Areas I and II delimited by line Λ = 1 refer to Table 2 in the main text. (0.95 MB TIF) [file pone.0002687.s005.tif]

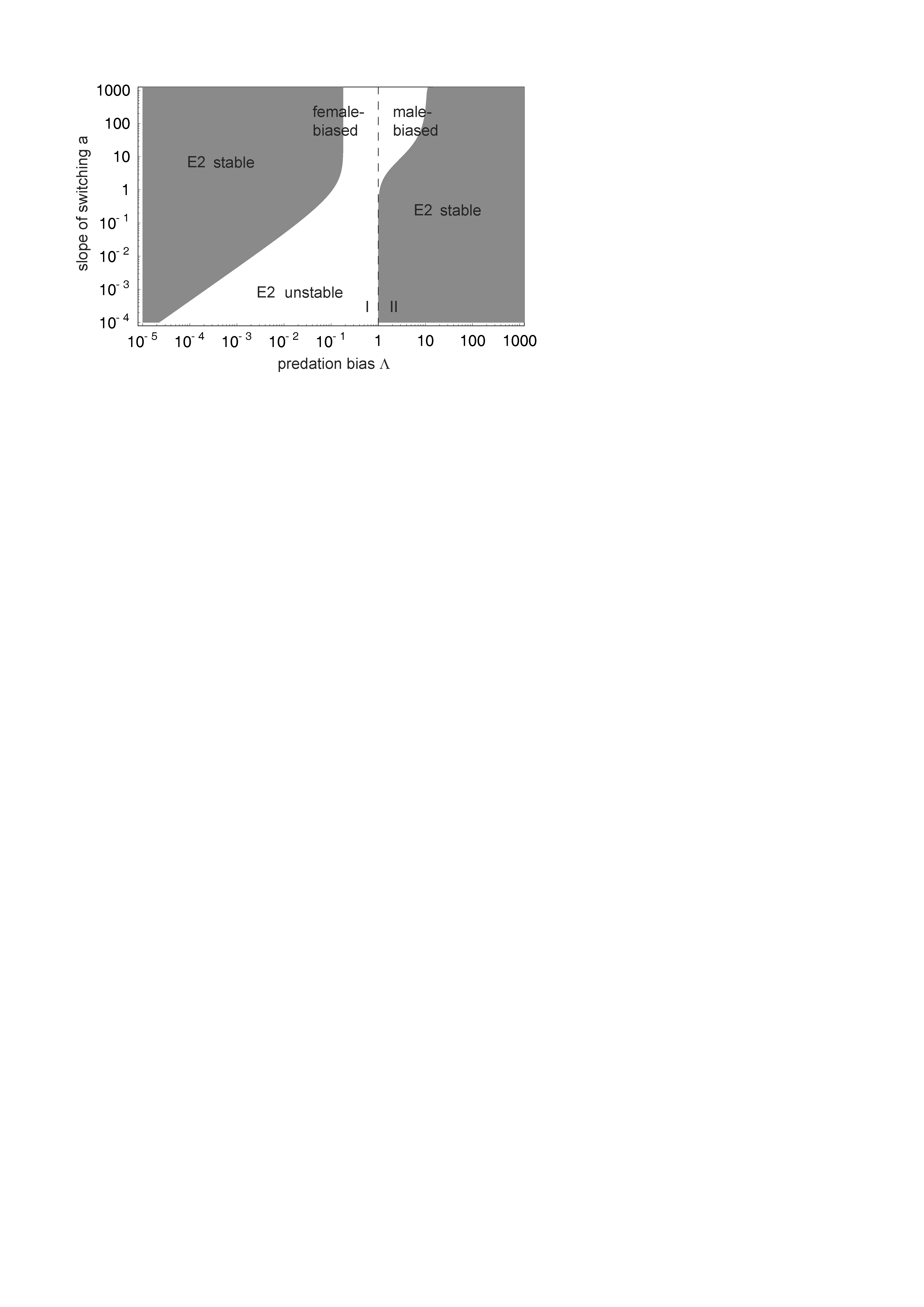

Supplement: Figure S3 — Stability of model (S2) in Text S2 with unlimited polygyny and no mate-finding Allee effect. Combined effect of predation bias and steepness in predator switching on the stability of the predator-prey equilibrium E 2 of model (S2). Parameters: b = 3, d = 0.2, Q = 0, e 1 = 0.2, e 2 = 0.1, and M = 1. E 2 is locally stable within the grey area. Areas I and II delimited by line Λ = 1 refer to Table 2 in the main text. (0.96 MB TIF) [file pone.0002687.s006.tif]

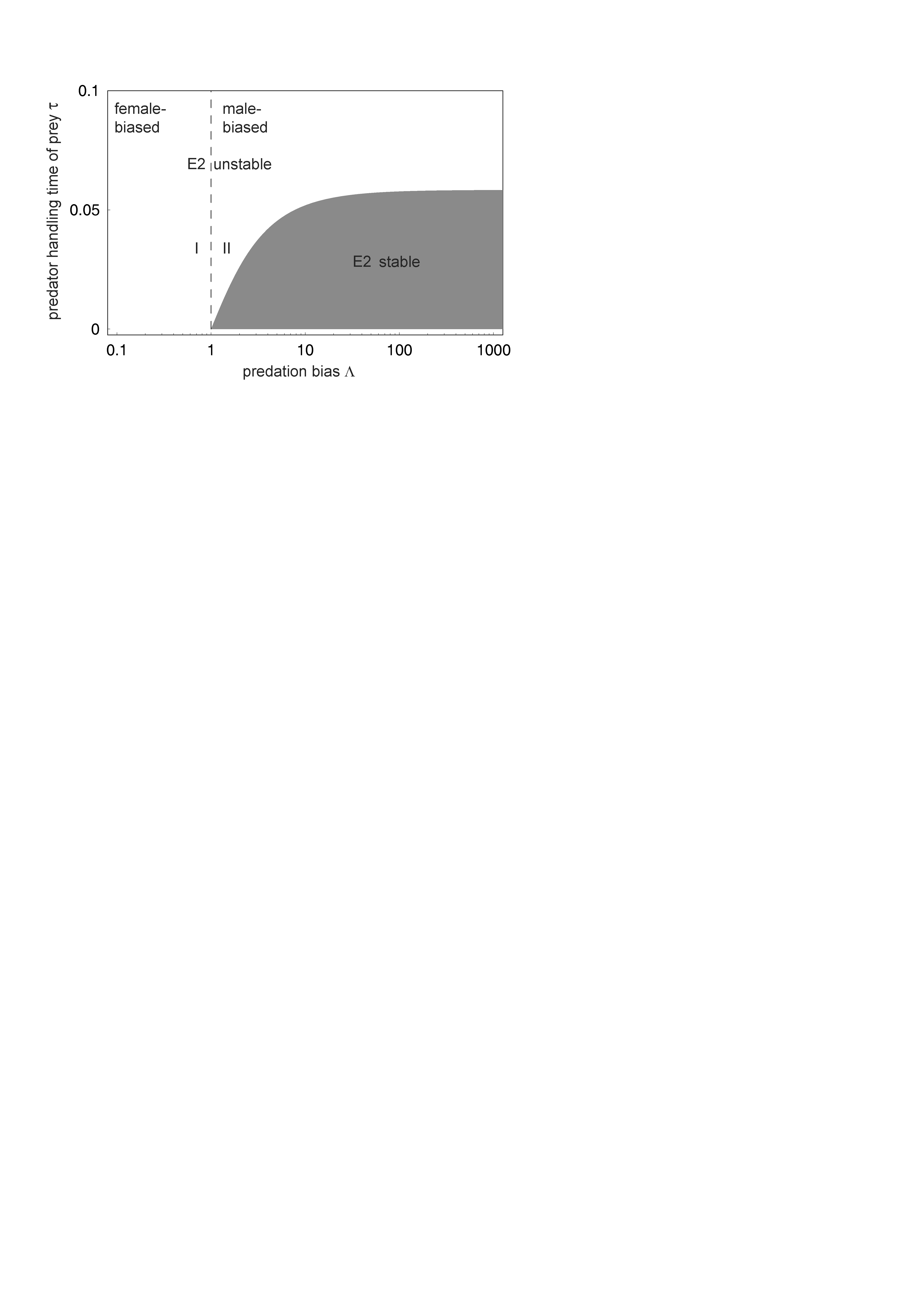

Supplement: Figure S4 — Stability of model (S3) in Text S2 with unlimited polygyny and no mate-finding Allee effect. Combined effect of predation bias and handling time of the predator with Holling type II functional response. Other parameters: b = 3, d = 0.2, Θ = 0, e 1 = 0.2, e 2 = 0.1, and M = 1. E 2 is locally stable within the grey area. Areas I and II delimited by line Λ = 1 refer to Table 2 in the main text. (0.93 MB TIF) [file pone.0002687.s007.tif]
